# Muscle Stem Cell Microenvironment and Functions in Muscle Regeneration

**DOI:** 10.3390/biom15060765

**Published:** 2025-05-26

**Authors:** Wenjing Li, Minyou Chen, Lingli Zhang

**Affiliations:** 1 College of Athletic Performance, Shanghai University of Sport, Shanghai 200438, China; 2421852039@sus.edu.cn; 2 School of Sport, Exercise and Health Sciences, Loughborough University, Loughborough LE11 3TU, UK; jingquinn001@gmail.com

**Keywords:** muscle stem cells, microenvironment, muscle regeneration, function

## Abstract

Muscle stem cells (MuSCs) are the key to muscle regeneration. The activation and maintenance of MuSCs require the precise regulation of their microenvironments. Myofibers and other cells including endothelial cells, fibroblasts, and immune cell populations constitute the cell components of the MuSC niche. The communication between these cell populations and MuSCs play an essential role in muscle repair. Furthermore, the physical and chemical stimulations around MuSCs also affect the cell behaviors of MuSCs. Extracellular matrix (ECM) and the factors stored in it generate a repair-promoting niche for efficient muscle regeneration. Understanding the mechanism of muscle stem cell regulation is the basis of clinically optimizing muscle repair. In this review, we discuss recent findings about the microenvironments of MuSCs and their functions in muscle regeneration, which would shed light on new targets and strategies for muscle injury treatment.

## 1. Introduction

Skeletal muscles contract and pull the attached bones under the control of nerves, using movable bone connections as hubs to produce lever movements. Skeletal muscle is essential to motion and metabolism. Aside from supporting body movement, it functions as a metabolic and endocrine organ, interacting with other organs and maintaining homeostasis through saving and secreting metabolites and factors [1]. Myofibers are the primary component of skeletal muscle, which are responsible for force production and the release of myokines. The arise of myofibers needs the differentiation of muscle stem cells (MuSCs), also called satellite cells. These cells are located beneath the basal lamina of muscle and are close to muscle fibers [2]. MuSCs are a critical reservoir for muscle regeneration under trauma and other challenges. Under homeostatic conditions, MuSCs are maintained in quiescence. With extrinsic stimuli, such as injury and exercise, MuSCs are activated, followed by proliferation and differentiation [3]. A stem cell niche is the microenvironment that facilitates and protects stem cell function and identity [4]. The multiple types of niche cells, extracellular matrix (ECM), and factors reserved in the ECM as well as the physical and mechanical signals constitute the microenvironment of MuSCs. These microenvironment factors change dynamically during development, aging, and regeneration. The spatiotemporal remodeling of the microenvironment is essential to regulate the cell behaviors of MuSCs and efficient muscle regeneration [5].

After muscle injury, the MuSCs need to transit from the quiescent state toward the activated state to start the proliferation and differentiation processes. Delayed activation impairs the repair efficiency, while excessive activation drives the exhaustion of the stem cell pool [6]. The microenvironment signals are responsible for pushing the MuSCs into the cell cycle and promoting their self-renewal at the same time [6]. The stem cell niche is also associated with the migration, fate determination, and heterogeneity of MuSCs [7,8,9].

In this review, we discuss the influence of niche factors, including niche cells, ECM, mechanical stimulation, and oxygen, on the cell behavior of MuSCs during muscle regeneration.

## 2. Characteristics and Heterogeneity of MuSCs

The inherent capacity for self-renewal over multiple cycles of injury and regeneration, giving rise to downstream differentiated cells, defines the concept of muscle stem cells [10]. Anatomically, myogenic stem cells were initially defined as cells occupying grooves or depressions between the basal lamina and sarcolemma of muscle fibers [11]. Subsequently, the marker gene expression of these cells, including CD34, m-cadherin, and Pax7, were reported [10,12]. Cells with this gene signature were then discriminated from their differentiated myoblasts, acting as committed stem cells to support muscle development and regeneration. The potent self-renewal and differentiation capacity of satellite cells was proven by the transplantation of a single intact myofiber into radiation-ablated muscles. The transplantation experiment revealed that a few satellite cells were able to generate over 100 new myofibers containing thousands of myonuclei [13]. The transcription factor Pax7 expresses in quiescent MuSCs and co-expresses with the marker MyoD in activated and differentiated MuSCs, while the self-renewed MuSCs maintain the single positive expression of Pax7 [14]. As the main marker for MuSCs, Pax7 is critical for MuSCs in homeostasis and injury of muscle. Mice lacking Pax7 showed significantly reduced fiber diameter and nuclei, with poor cell survival [15]. Pax7 deficiency led to cell cycle arrest of MuSCs and myoblasts and dysregulation of myogenic regulatory factors (MRFs) [16,17]. Precise regulation of the expression and modification of Pax7 is the precondition for the normal function of MuSCs [18]. The unique expression pattern of Pax7 in MuSCs makes it an outstanding target for labeling MuSCs in reporter mice [15,19]. The direct label of Pax7+ MuSCs can be realized by the insertion of fluorescence proteins, such as ZsGreen and EYFP, in the Pax7 locus. Pax7-Cre or Pax7-CreERT2 mice allow for flexibility to tune the reporter protein and label period according to the research goal by combining with different reporter mice [19]. The fluorescence label of MuSCs not only allows for the isolation of these cells from the reporter mice, but also for imaging and real-time observations of the cell behavior of MuSCs. The advantages of the reporter mice supported by the specificity of Pax7 are apparent, but the limitation is that these cells cannot be obtained in wild type mice or human samples, which is why the combination of cell surface markers were built. After the avoidance of endothelial cells (CD31+), hematopoietic cells (CD45+), and mesenchymal stem cells (SCA1+), quiescent or activated MuSCs of adult mice are isolated by the marker VCAM1 [20]. Panels include other surface markers recognizing satellite cells such as integrin-a7, CD34, etc. [21].

The heterogeneity of the satellite cell pool has attracted the attention of researchers since the identification of the cells. Through clonal multicolor lineage tracing, MuSCs were found to maintain their clonal complexity during aging to allow the aged muscle to respond to injury. However, repeated injury and repair decreases the clonal complexity. These findings suggest distinct regulations of the satellite cell populations during aging and regeneration [22]. The development and progress of single-cell multi-omics are driving the investigation of the population heterogeneity and identification of new markers of MuSCs. More populations of MuSCs have been defined with different properties in varying situations. MX1 was proposed to mark a population of skeletal stem cells with the other marker αSMA. These skeletal stem cells specifically express CCR3 and CCR5 to promote cell migration and bone regeneration [23]. In skeletal muscles, MX1 was demonstrated to mark a subset of MuSCs capable of expansion under stress. These MX1+ cells minimally contribute to endogenous muscle regeneration because of the low abundance. Intriguingly, these cells exhibited dramatic clonal expansion upon radiation stress, showing reserve stem cell properties [23]. CD34 was found to be able to distinguish genuine MuSC subpopulations (CD34^High^) and cells committed to myogenic differentiation (CD34^low^) [24]. The Gli family of transcription factors are pivotal Hedgehog signaling effectors. Gli1 has been found to mark a population of osteoprogenitors for bone formation and fracture repair [25]. Recently, a study showed that Gli1+ identified a sentinel MuSC for muscle regeneration. Gli1+ MuSCs have stronger mTOR signaling activity, more mitochondria compared with Gli1-MuSCs, and actively contribute to muscle regeneration [26]. This research revealed the metabolic heterogeneity of MuSC populations. The heterogeneity of the satellite cell pool also presents in human muscles. A study combining single-cell RNA-Seq and flow cytometry identified and separated new subpopulations in Pax7+ human MuSCs. They pointed out a population marked by the combination of CXCR4/CD29/CD56/CAV1+ that showed resistance to activation and enhanced engraftment after transplantation compared with CAV1-MuSCs [27]. More exquisite and detailed analyses of mouse and human cell populations provided researchers with the opportunity to propose evidence of MuSC heterogeneity and identify subpopulations with greater regenerative capacity. Nonetheless, the mechanism of how heterogeneity forms in development and regeneration still remains to be further interrogated.

**Figure 1:**
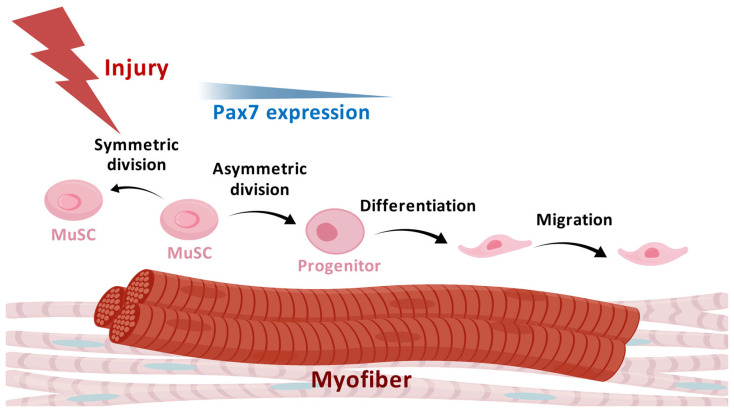
Cell behaviors of MuSCs after muscle injury. MuSCs go through symmetric division to maintain the stem cell pool. On the other hand, the differentiation and migration of MuSCs are needed to give rise to the cells for muscle regeneration. (Created with BioGDP.com [37]).

Maintenance of the quiescence state of MuSCs is necessary to prevent the activation and exhaustion of the stem cell pool [28]. During muscle growth and regeneration, the MuSCs are stimulated by the coordinated intrinsic and extrinsic signals and transition to the activation state [29,30]. To support the formation of new myofibers after injury and the growth of pre-existing myofibers upon mechanical stimulations, the activated MuSCs go through processes including proliferation, differentiation into myoblasts, fusion of the differentiated myocytes, and the formation of myofibers [31]. During proliferation, the satellite cells undergo symmetric division and asymmetric division, giving rise to two stem cells or one differentiated cell and one stem cell, respectively [28,32]. Planar symmetric division, in which the division direction is parallel to the basal lamina, expands the satellite cell pool. Through utilizing the reporter directed by Myf5 Cre in the mouse model, the asymmetric division generated one Myf5-stem cell and one Myf5+ progenitor that could be observed [32]. The balance between symmetric and asymmetric division is essential for maintenance of the stem cell pool and the necessary regeneration capacity. During regeneration, some MuSCs may directly go through asymmetric division to give rise to committed progenitor cells [33]. Signaling pathways including epidermal growth factor (EGF), mitogen-activated protein kinase (MAPK), and protease-activated receptors (Par) have been proposed to regulate the division of satellite cells [34]. The symmetric division of satellite cells is critical to self-renewal, regulated by multiple microenvironmental factors (discussed later). The myogenic differentiation of MuSCs is regulated by PAX3, PAX7, and MRFs [33]. Pax7-deficient adult mice showed the complete absence of residing satellite cells in the muscle [15]. Pax3 expression leads to the activation of MyoD and maintenance of Myf5, which results in myogenic determination and myogenesis [35]. The migration of MuSCs is required for efficient muscle regeneration, which is regulated by intracellular signals and niche factors [8,9,36]. The cell behaviors of MuSCs in muscle regeneration are summarized in Figure 1.

## 3. Microenvironment of MuSCs and Their Regulation in Muscle Regeneration

There are two major waves of muscle formation: embryonic and fetal myogenesis. During the process of embryonic myogenesis, almost all skeletal muscles, except for the craniofacial muscles, are derived from the paraxial mesoderm [38]. The epaxial dermomyotome gives rise to the deep back muscles whereas the hypaxial extremity of the dermomyotome gives rise to the rest of the musculature [39]. The delamination and migration from the somite toward the limb buds of myogenic progenitor cells initiate the formation of a myotome, which is a layer of muscle derived from the fused differentiated myocytes [40]. In the late stage of embryonic development, PAX3/PAX7+ cells from the central dermomyotome develop into almost all of the satellite cells [41]. The following fetal myogenesis occurs with the differentiation of PAX7+ cells into myoblasts. The fusion of the myoblasts and the fibers formed during embryonic myogenesis support muscle growth [42].

### 3.1. The Cell Components of the MuSC Niche

**Figure 2:**
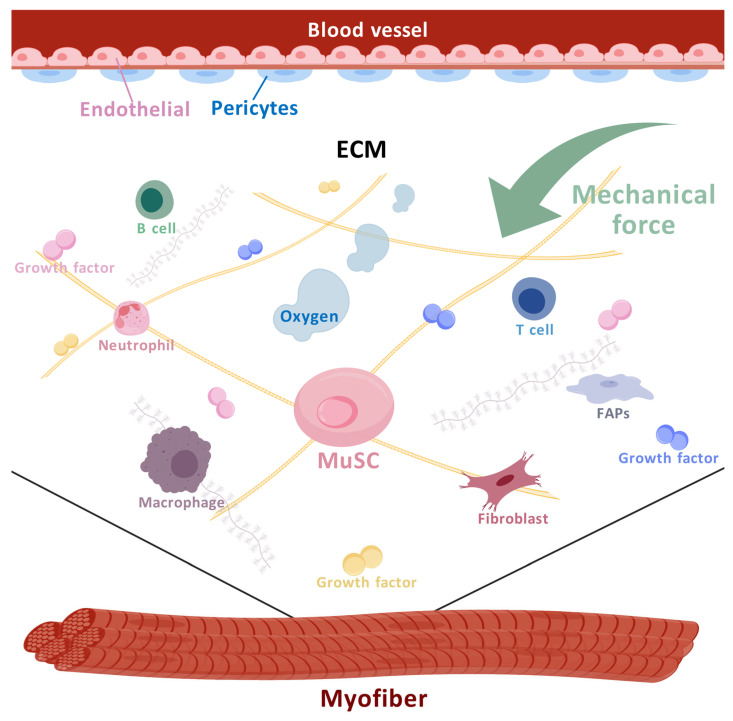
Microenvironments of MuSCs. Cells including myofibers, endothelial cells, pericytes, FAPs, fibroblasts, and immune cells, etc. generate the cellular niche of MuSCs. Non-cellular factors such as the ECM, mechanical signals, and oxygen also contribute to the microenvironment of MuSCs. (Created with BioGDP.com [37]).

The cells’ niche of MuSCs is composed of myofibers, endothelial cells, pericytes, fibro adipogenic progenitors (FAPs), fibroblasts, and immune cells [43]. The cellular and non-cellular microenvironments of MuSCs are depicted in Figure 2.

#### 3.1.1. Myofibers

Myofibers can influence the cell behavior of MuSCs from a few aspects. The deformation induced by the stretch of myofibers is a mechanical signal to MuSCs. Except that in the intact fibers, protein and metabolites stored in the myofibers that are released from the broken myofibers when injury happens would also affect MuSCs in muscle regeneration [44,45,46,47]. Moreover, the death and damage of myofibers triggers a cascade of release including responsive proteins and metabolites. These mediators play crucial roles in regulating MuSCs during muscle inflammation and regeneration. Due to the varied forms of cell death and damage of myofibers, their influence on MuSCs is diverse [48].

Generally, muscle fibers are typologically divided into fast-twitch fibers, or type II fibers, and slow-twitch fibers, or type I fibers [49]. Fast-twitch fibers are glycolytic, which are able to generate more power, whereas slow-twitch fibers are relatively more oxidative and fatigue-resistant [50,51,52]. There is evidence that shows that the satellite cells in slow- and fast-twitch muscles display different properties. Fast-twitch fibers derived from satellite cells were found to have a higher proliferation efficiency but lower self-renewal potential [53]. These observations are consistent with the discovery of the faster regeneration of injured fast-twitch muscles and the more difficult reconstruction and fibrosis development of slow-twitch muscles [54]. In this research, increased and prolonged inflammation were found in slow-twitch muscles. Inflammation response is a key regulator of tissue repair. The mechanism of how the inflammation response in different fibers lead to different repair efficiency still remains to be investigated.

#### 3.1.2. Other Cellular Components of the MuSC Niche

Endothelial cells constitute the inner cellular lining of blood vessels, and the monolayer of endothelial cells is called the endothelium. Pericytes are mural cells surrounding the endothelium. These vascular cells with a distinct and complicated gene signature are a part of the MuSC microenvironment [55,56]. FAPs are believed to give rise to the accumulated fatty and fibrotic tissues in aged and pathological skeletal muscles. These multipotent cells, which are also able to differentiate into chondrocytes and osteoblasts, actively participate in muscle regeneration through affecting MuSCs (discussed later) [57]. Fibroblasts are regarded as the main ECM producing cells in skeletal muscles [58]. The ECM and growth factors secreted by fibroblasts are necessary for the building and maintenance of tissue structure and the function of muscles. However, an excess accumulation of ECM in injury or pathological situations would lead to muscle weakness and impair muscle function [59].

An intriguing study found that the reprogrammed myofibers acted as niche regulators to induce the activation of MuSCs and accelerate muscle regeneration in young mice [47]. Through the direction of the Acta1-Cre mouse tool, the researchers inducibly overexpressed OSKM (Oct3/4, Sox2, Klf4, and c-Myc) specifically in myofibers to partially reprogram the myofibers in vivo. The alteration of myofibers enhanced the proliferation but not the self-renewal of MuSCs. What is interesting about this research is the OSKM induction in MuSCs by Pax7-CreER had no influence on muscle regeneration, which demonstrated the indispensable role of myofibers as a niche factor of MuSCs [47]. As a form of programmed death, the necroptosis of myofibers can also influence MuSCs through releasing factors. The temporary expression of tenascin-C, controlled by the necroptosis of myofibers, facilitates MuSC proliferation through EGFR signaling [60]. Another study co-culturing mechanically damaged myofibers and MyoD+ satellite cells found that damaged-myofiber-derived factors (DMDFs) induced the satellite cells to enter the cell cycle [46]. Specifically overexpressing PCG-1α in myofibers by Mck-Cre resulted in increased integrin-α7+ myogenic progenitors. Although the increased myogenic progenitors did not accelerate muscle regeneration, the cells were more prone to myogenesis instead of adipogenesis, proposing that myofibers affect the fate commitment of myogenic progenitors [61]. A detailed study utilizing real-time intravital imaging demonstrated that the spatial location of the damaged myofibers affected the migration directionality of myogenic progenitors [62]. It is now clear that while the myofiber-derived factors are able to regulate the cell behaviors of MuSCs, more specific factors remain to be identified. Endothelial cells and muscle cells share common embryonic progenitors. Interestingly, the appearance of the endothelial cell signature was proven to promote the survival of MuSCs via the VEGFA-FLT1-AKT1 signaling pathway [63]. Although not directly targeting MuSCs, the lactate released by endothelial cells constructed a repair-promoting niche in skeletal muscle by inducing the polarization of M2 microphages. The polarized macrophages then promoted the proliferation and fusion of myogenic progenitors, leading to the enhanced repair of muscle after ischemia-revascularization injury [64].

FAPs are the main source of intramuscular adipose tissue. The role of these cells in muscle regeneration has drawn attention for a long time. It has been found that exercise reduced FAPs and adipose formation in injured muscle through musclin [65]. In this study, FAPs could be harmful to muscle homoeostasis. However, FAPs can also generate a favorable niche for satellite cells in muscle regeneration. FAP-like cells were found to be released from the subcutaneous adipose tissue and infiltrate to the damaged muscle. Inhibition of the infiltration of these cells impaired muscle regeneration, revealing the dependency of the muscle niche on the heterogeneity of FAPs [66]. FAPs are capable of communicating with MuSCs via the secretion of extracellular vesicles (EVs). The miRNAs in these EVs mediate the crosstalk between FAPs and MuSCs and push muscle regeneration. Among the miRNAs, miR-127-3p has been proposed to be the most abundant, which activates myogenesis through targeting the sphingosine-1-phosphate receptor, S1pr3, on MuSCs. In the other direction, miRNA containing EVs from MuSCs act as negative regulators of adipose tissue infiltration [67]. FAPs themselves are able to differentiate toward myofibroblasts and produce excessive collagen, leading to fibrosis and the hindered regeneration of muscle. A deficiency of mammalian Ste20-like kinase 1/2 (MST1/2) in FAPs promoted the differentiation of FAPs toward fibroblasts and impaired the proliferation and differentiation of myoblasts [68]. An unexpected study analyzed the single-cell RNA-Seq data of limb muscles at successive developmental stages and found that a population of fibroblasts contributed to muscle development through fibroblast-to-myoblast conversion. The conversion of the lateral plate mesoderm-derived cells to myogenic cells regulated by BMP signaling was to support the normal structure of muscles [69].

In the study of skeletal muscle regeneration and repair, different models are utilized to observe the process of regeneration. The commonly used approaches include the injection of cardiotoxin (CTX), notexin (NTX), barium chloride (BaCl_2_), freeze injury, ischemia, and volumetric muscle loss [70,71,72]. Since these methods may stimulate different injury response muscles, the method used to induce muscle injury should be taken into account when comparing the results of different research articles [72].

#### 3.1.3. Immune Cells

The immune system refers to the coordination of immune cells, the factors they secrete, and the process of protecting tissues. Antigen-independent innate immunity and antigen-dependent adaptive immunity involve different immune cell populations. In innate immunity, a limited range of immune cells are responsible for detecting and resolving a wide range of pathogens. After tissue injury or the invasion of pathogens, innate immune cells can expand and be recruited to the injury site to initiate inflammation and the sequential repair processes. The cells involved in innate immunity are macrophages, neutrophils, dendritic cells, mast cells, basophils, eosinophils, natural killer cells, and innate lymphoid cells [73]. On the other hand, adaptive immune cells include antigen-specific T cells and the antibody-producing B cells. Upon antigen presentation process stimulation, T cells differentiate into cytotoxic T cells (CD8+) and T-helper cells (CD4+). The regulatory T cells (T reg) are a subset of CD4+ T cells [74]. CD8+ cytotoxic T cells are mainly involved in the destruction of infected cells and killing tumor cells expressing certain antigens while the CD4+ T-helper cells primarily function to augment the immune response [75].

The microenvironments defined by immune cells in skeletal muscles can be very different between homeostasis and injury conditions because of the inflammatory response after muscle injury and the participation of immune cells in muscle regeneration and remodeling. Neutrophils and macrophages are phagocytes that appear at the injury site in the earliest stage to remove cellular debris through phagocytosis [76]. Neutrophils can recruit macrophages to the injury site through secreting chemokines like TNF-α, IL-1, and IL-8 [77]. In skeletal muscles, resident macrophages function as sensors of pathogens and injury, while the recruited macrophages amplify the inflammatory response [78]. Except for phagocytosis, macrophages also function to secrete cytokines and growth factors, antigen presentation, and recruit immune cells [79].

Macrophages are tightly associated with the initiation and resolution of the inflammation during tissue regeneration. The classic category of macrophages divides them into pro-inflammatory M1 macrophages and anti-inflammatory M2 macrophages [80]. However, with further in-depth investigation of the population characteristics and function, the concept of macrophage population is continuously updating, and our understanding of the versatility of macrophages has been expanded. Furthermore, there is accumulating evidence utilizing intravital and real-time imaging to capture the fast cell behaviors of these phagocytes, showing that they also play other important roles in regulating MuSCs, which we will discuss in the next section.

In a study utilizing the volumetric muscle loss injury model, which involves significant muscle damage and removal, the interaction and population balance between neutrophils and natural killer cells were found to be critical to muscle regeneration [81]. Besides the rapid recruitment of natural killer cells, there was a persistence of neutrophils over 2 weeks after volumetric muscle loss injury, which impaired the regenerative capacity of MuSCs through suppressing the fusion process. Mechanistically, they proposed that blocking transforming growth factor beta 1 (TGFβ1) signaling could reduce the accumulation of neutrophils and fibrosis of the repaired muscles [81]. The communication network between these immune cell populations is highly complicated and dynamic. More cell population datasets are waiting to be analyzed to reveal the influence of the immune microenvironment on MuSCs. During the inflammation response and tissue regeneration, the appearance and disappearance of immune cells need to be accurately regulated. Not only does the persistence of neutrophils hinder muscle regeneration, but the deficiency of neutrophil infiltration also leads to an impaired repair outcome. As the key sensor of muscle injury, the regulation of neutrophil infiltration can be associated with multiple other immune cell populations. The following report emphasized the importance of platelets, where in mice with Cxcl7-knockout platelets, the infiltration of neutrophils in injured muscle was compromised, resulting in reduced myofiber size and muscle strength [82]. Furthermore, the exacerbated inflammation after the compromised neutrophil infiltration in the injured muscle is noteworthy and waits to be elucidated. Utilizing a mouse and human long-term exercise training model, one study showed that the maturation marker expression in neutrophils increased after the exercise stimulation, and the activated neutrophils improved the performance of muscle cells in the wound healing assay [81]. Given that skeletal muscles are highly active tissues in exercise training, the influence of exercise on the immune microenvironment of MuSCs is worthy of further exploration.

**Figure 3:**
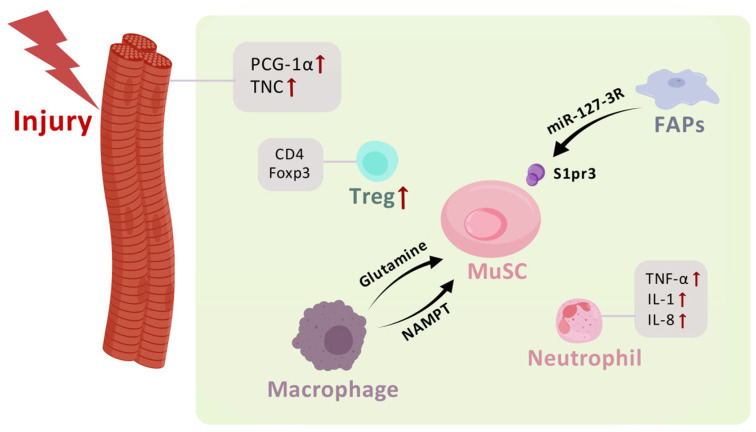
The factors in the microenvironment that regulate cell behaviors of MuSCs. Treg, regulatory T cell. (Created with BioGDP.com [37]).

Macrophages play a versatile role in homeostatic and injured skeletal muscle [78]. Due to the plasticity and population diversity, the role of macrophages in regulating tissue repair has been reported in tissues including skin, liver, and heart [83,84,85]. In injured muscle, macrophages are crucial to the function of MuSCs by generating a repair-promoting niche. From a metabolic perspective, the researchers noticed that injured and aged muscle underwent intra-tissue restrictions of glutamine. In macrophages with glutamate dehydrogenase 1 (GLUD1)-deficiency, the activity of glutamine synthetase and the release of glutamine were enhanced. The increased glutamine released from the macrophages was accepted by the MuSCs, leading to promoted proliferation and differentiation of the MuSCs and improved muscle regeneration [86]. In the muscle injury model of zebrafish, macrophages were found to provide a transient muscle stem cell niche through the release of the cytokine nicotinamide phosphoribosyltransferase (NAMPT). Utilizing single-cell RNA-Seq analysis of the macrophage populations, the researchers identified a subpopulation of macrophages in the injured muscle of zebrafish that dwell within the injury to generate the repair niche. The cytokine NAMPT promotes the proliferation of MuSCs via the C-C motif chemokine receptor type 5 (CCR5) expressed by MuSCs [87]. Zebrafish, as the research model, was convenient for the long-term, real-time, and intravital observation of multiple populations so that the transient repair niche provided by the subpopulation of macrophage could by captured and investigated. Taking advantage of three-dimensional time-lapse imaging on live reporter mice, the spatiotemporal interaction between the myeloid cells and satellite cells was found to be transient, similar to the phenomenon observed in zebrafish. Moreover, the proliferation of satellite cells required macrophages, but the constant contact with macrophages was not necessary [88]. Using experiments including parabiosis, lineage tracing, and single-cell RNA-Seq, one study identified three populations of skeletal muscle-resident myelomonocytic cells, TIM4 and LYVE1 positive macrophage, LYVE1-TIM4-macrophages, and CD11C and MHCII positive dendritic cells. The TIM4 negative macrophages were replenished from blood, whereas the TIM4 positive macrophages were locally self-renewed. These circulation-derived and residential self-renewed macrophages exert a multifaceted function in skeletal muscles including clearing damaged-induced apoptotic cells and regulating muscle fiber composition [89]. Aside from the chronic and acute injury related tissue specific macrophage populations, age-associated functional macrophage populations using the markers LYVE1 and MHCII have also been found [90]. The use of single-cell transcriptome analysis is pushing more and more subpopulation identification in macrophages. In the current stage, the investigators are still constructing the initial knowledge of different populations of macrophages in skeletal muscles under varied conditions. The relationship between these populations and how these cell populations communicate with MuSCs remain to be uncovered in further research. Single-cell RNA-Seq and flow cytometry analysis in mice demonstrated that lymphoid cells accounted for about 12% of the immune cells in young homeostatic skeletal muscle [91]. The proportion increased to about one third during the aging of skeletal muscle. In T cells sorted from old mice, the expression of genes associated with pro-inflammatory activation, cytotoxicity, and exhaustion increased [91]. The dynamic immune niche provided by lymphoid cells and their function on MuSCs are still waiting to be uncovered. A unique population of CD4+Foxp3+ Treg cells were found to accumulate in the injury site shortly after injury. Ablation of the Treg cells compromised muscle repair. Amphiregulin (Areg), a growth factor of the EGF family, was identified as being released by these muscle Treg cells to promote the proliferation of myogenic progenitors [92]. Impaired muscle regeneration was also found in mice with CD8α deficiency. The deficiency of CD8α resulted in the decrease in macrophage recruitment and satellite cell number [93]. CD4+ Foxp3+ T reg cells were required for muscle regeneration. These CD4+ Foxp3+ T reg cells were recruited to the injury site by FAP-derived IL-33 to promote muscle regeneration [94] (Figure 3, Table 1).

### 3.2. Non-Cellular Niches

The non-cellular microenvironments of MuSCs include, but are not limited to, the ECM, the factors stored in the ECM, mechanical signals, metabolites, and oxygen.

#### 3.2.1. ECM and Factors

The ECM functions not only as a network-like scaffold to connect the cells, but also as a mediator of extracellular biochemical and physical signals, such as growth factors, cytokines and mechanical stimulations, to regulate cell behaviors [95,96,97]. Collagen is the main ECM protein and the most abundant protein in mammals, accounting for about 30% of the total protein mass [98]. In skeletal muscles, collagens, especially types I and III collagens in the interstitial connective tissues, are the main ECM components, while types IV and VI are the main collagens in the basement membrane [99]. There are many other proteins, including fibronectin (FN), laminin, etc., in the extracellular space besides collagens [100,101]. The multi-modular structural feature of these extracellular proteins allows them to bind with extracellular collagens, growth factors, and elements expressed on the cell membrane such as integrins [99,102]. One of the important functions of the ECM is the storage and release of multiple growth factors, cytokines, chemokines, and enzymes to exert the temporal and spatial regulation of cell behavior and communication [103]. Through tuning the mechanical properties of the proteins surrounding the cells, the ECM conveys different mechanical signals and triggers intracellular mechanotransduction [104]. Mechanical studies examining the modulus, which refers to the stiffness or elasticity, of muscle samples have reported that the modulus of muscle fiber bundles can be twofold higher than that of a single fiber, suggesting the significance of the ECM in maintaining the normal mechanical property of muscles [105,106].

The lack of collagen VI in mice compromised muscle regeneration and reduced the self-renewal capability of satellite cells. In vitro and in vivo experiments proved that collagen VI was able to enhance the maintenance and survival of PAX7 positive cells through regulating the mechanical property of muscle tissue [107]. Collagen VI acted not only as the structural component of ECM to provide the appropriate mechanical niche for MuSCs, but also regulated the stemness and differentiation of MuSCs as a soluble ligand, which avoided the early differentiation of MuSCs and preserved their stemness feature [108]. The surface marker CD34 was proposed to distinguish the genuine (CD34 high) and primed state (CD34 low) of MuSCs [23]. The niche factor IGF1 promoted the genuine-to-primed conversion of stem cells, causing the regenerative failure of muscle [24].

Laminin is the major component of the basement membrane, which functions as a key structural and niche factor for muscle cells [109]. The stemness of the multipotent PDGFβ+ cells residing in muscles is regulated by laminin. Deficiency of laminin in PDGFβ+ cells causes muscular dystrophy. During muscle regeneration, these PDGFβ+ cells secrete laminin to inhibit their proliferation and adipogenesis via gpihbp1 as well as promote the myogenesis of PDGFβ+ cells [110]. Upon activation, satellite cells build a niche that is beneficial to their expansion and self-renewal through regulating the remodeling of the ECM and the deposition of laminin-α1 and laminin-α5 into the basal lamina [111]. During the process of muscle regeneration, laminins are remodeled by matrix metalloproteinase, which results in the changes in the spatial distribution of the isoforms of laminins [111]. The expansion and self-renewal of satellite cells are compromised if the remodeling of the ECM is prevented by the blocking of matrix metalloproteinase. Mechanistically, laminin-α1 promotes the proliferation and self-renewal of MuSCs through integrin-α6, which is upregulated in activated satellite cells [111]. In a study observing the interaction between satellite cells and ECM using acellular ECM myoscaffolds that recapitulated the ECM architecture and composition, it was found that the laminin deposition in dystrophic muscle-derived myoscaffolds impaired the remodeling and adhesion of muscle progenitor cells. The differentiation of muscle progenitor cells required laminin remodeling [112].

#### 3.2.2. Mechanical Signals and MuSCs

Intrinsic and extrinsic mechanical signals regulate cell behaviors and fate commitment [113,114,115]. Skeletal muscles are the primary active force producing tissue in the body [116]. MuSCs are influenced by mechanical stimulations from the surrounding myofibers and other cells as well as the ECM. Through cell–cell and cell–ECM adhesion, the mechanosensitive ion channels expressed in their cell membrane and the cilium, the stem cells sense the mechanical stimulations [117].

Using mT/mG double-fluorescent Cre-reporter mouse and multiphoton microscopy, one study observed how the stretch of the myofiber induced the tensile and shear deformation of MuSCs [45]. The deformation of MuSCs changed their proliferation and differentiation related gene expression with increased mRNA levels of c-Fos, Cdk4, IL-6, and upregulated ERK1/2 and p38 MAPK signaling. In vivo research found that compression stimulation was able to drive activated MuSCs back to a quiescent state through the upregulation of Notch signaling [118]. The mechanosensation process is critical for the cell behavior regulation of stem cells in tissue regeneration. In muscles, the mechanosensitive calcium ion channel PIEZO1, mediating the mechanotransduction of satellite cells, is required for efficient muscle regeneration. Through the morphological investigation and classification, the research reported the heterogeneity of MuSCs according to different patterns of cellular protrusions and proposed three states of quiescent satellite cells: responsive, intermediate, and sensory. Intriguingly, PIEZO1 played a key role in the state shift of these cells, which promoted muscle regeneration. Activation of PIEZO1 shifted the cells to more responsive cells, whereas a deficiency of PIEZO1 shifted the cell distribution to a less responsive state, which is similar to the phenotype in dystrophic muscles [119]. PIEZO1 was also required for the proliferation and regenerative function of MuSCs in myogenesis, which was associated with PIEZO1-Rho signaling [120].

#### 3.2.3. Oxygen Tension and MuSCs

The concentration of oxygen is also one of the determinant factors that define the development and differentiation of stem cells through metabolic regulation. As a critical component of the stem cell niche, oxygen tension influences the process of embryonic morphogenesis, tissue homeostasis maintenance, and tissue regeneration [121]. The partial pressure oxygen of inspired air decreases from 21% to 2–9% as it enters the lung and travels in the blood throughout the body [122]. Depending on the vascular supply, even in the same tissue, the oxygen pressure at different locations varies. Sites with oxygen pressure that are relatively lower than other surrounding sites are considered as a hypoxia microenvironment. The benefit of hypoxia in maintaining the undifferentiated state of stem cells has been reported in hematopoietic, mesenchymal, and neural stem cells [121].

In vitro experiments found that a hypoxic culture condition upregulated the expression of Pax7 while it downregulated the expression of MyoD and myogenin in the satellite cell-derived myoblasts [123]. Besides facilitating the maintenance of the quiescent state and self-renewal capability of MuSCs, the hypoxic environment also participates in the regulation of MuSC fate commitment, influencing the adipogenic differentiation tendency [124]. Furthermore, the effects of hypoxia with the same concentration of oxygen may be different on cells from muscles with distinct metabolic properties. Compared with stem cells derived from the semimembranosus muscle, cells from the diaphragm showed a greater myotube formation response in the stimulation of a 5% oxygen concentration [124]. This means that although hypoxia provides a quiescence-maintaining niche for MuSCs, the culture protocol should be customized for cells with specific origins. The hypoxic microenvironment resulting from muscle injury also causes the osteogenic differentiation of multipotent muscle stromal cells, leading to heterotopic ossification in muscle regeneration [125].

## 4. Conclusions and Perspectives

Revealing the regulation of MuSCs is the basis of optimized muscle repair. The coordination between the ECM, neighboring niche cells, and the mechanical environment orchestrates MuSC quiescence, activation, proliferation, and differentiation. Disruptions in the microenvironmental harmony will profoundly impair the regenerative capacity of muscles, highlighting the niche as a potential therapeutic target. Looking ahead, understanding how MuSCs respond to complex signals will require more in vivo models and real-time tracking technologies. Furthermore, more translational and clinical research is also necessary to develop new treatment strategies in the treatment of muscle trauma.

Due to the technological development of single-cell multi-omics, there has been an explosion of knowledge regarding cell populations and the identification of stem cell markers in tissues including skeletal muscle. However, a comprehensive characterization of the cell populations of FAPs and fibroblasts is still lacking. Given the complexity of these cell populations, it is unfeasible to label or isolate the different FAP or fibroblast cell populations to observe their communication with MuSCs. Similarly, the uncovering of the heterogeneity of MuSCs and the relationship between these different populations is still in the early stage. As for immune cell populations, the cell composition alteration during muscle aging has attracted broad attention. How these alterations interact with the satellite cell subpopulations remains to be interrogated.

Metabolites are a potential niche factor that regulates MuSCs. As above-mentioned, metabolites and metabolic enzymes released by macrophages affect MuSCs and muscle regeneration. Skeletal muscles are highly responsive to exercise. Recently, the lactylation in muscle has been noted [126]. The accumulation of lactate and other metabolites in muscles and their niche effects on MuSCs still remain to be revealed.

## Figures and Tables

**Table 1:** Niche factors and their effects on MuSCs.

Factor	Effects
PCG-1α	Increased myogenic progenitors
TNC	Promoted MuSC proliferation
Glutamine	Promoted MuSC proliferation and differentiation
NAMPT	Promoted MuSC proliferation
mir-127-3R	Activated myogenesis

## Data Availability

Not applicable.

## References

[1] Severinsen M.C.K., Pedersen B.K. (2020). Muscle-Organ Crosstalk: The Emerging Roles of Myokines. Endocr. Rev..

[2] Peault B., Rudnicki M., Torrente Y., Cossu G., Tremblay J.P., Partridge T., Gussoni E., Kunkel L.M., Huard J. (2007). Stem and progenitor cells in skeletal muscle development, maintenance, and therapy. Mol. Ther..

[3] Zeng W., Yue L., Lam K.S.W., Zhang W., So W.K., Tse E.H.Y., Cheung T.H. (2022). CPEB1 directs muscle stem cell activation by reprogramming the translational land. Nat. Commun..

[4] Zhu H., Lin X., Diao Y. (2021). Function and regulation of muscle stem cells in skeletal muscle development and regeneration: A narrative review. J. Bio-X Res..

[5] Loreti M., Sacco A. (2022). The jam session between muscle stem cells and the extracellular matrix in the tissue microenvironment. NPJ Regen. Med..

[6] Rodgers J.T., King K.Y., Brett J.O., Cromie M.J., Charville G.W., Maguire K.K., Brunson C., Mastey N., Liu L., Tsai C.R. (2014). mTORC1 controls the adaptive transition of quiescent stem cells from G0 to G(Alert). Nature.

[7] De Micheli A.J., Laurilliard E.J., Heinke C.L., Ravichandran H., Fraczek P., Soueid-Baumgarten S., De Vlaminck I., Elemento O., Cosgrove B.D. (2020). Single-Cell Analysis of the Muscle Stem Cell Hierarchy Identifies Heterotypic Communication Signals Involved in Skeletal Muscle Regeneration. Cell Rep..

[8] Brondolin M., Herzog D., Sultan S., Warburton F., Vigilante A., Knight R.D. (2023). Migration and differentiation of muscle stem cells are coupled by RhoA signalling during regeneration. Open Biol..

[9] Li C., Vargas-Franco D., Saha M., Davis R.M., Manko K.A., Draper I., Pacak C.A., Kang P.B. (2021). Megf10 deficiency impairs skeletal muscle stem cell migration and muscle regeneration. FEBS Open Bio.

[10] Seale P., Asakura A., Rudnicki M.A. (2001). The potential of muscle stem cells. Dev. Cell.

[11] Mauro A. (1961). Satellite cell of skeletal muscle fibers. J. Biophys. Biochem. Cytol..

[12] Beauchamp J.R., Morgan J.E., Pagel C.N., Partridge T.A. (1999). Dynamics of myoblast transplantation reveal a discrete minority of precursors with stem cell-like properties as the myogenic source. J. Cell Biol..

[13] Collins C.A., Olsen I., Zammit P.S., Heslop L., Petrie A., Partridge T.A., Morgan J.E. (2005). Stem cell function, self-renewal, and behavioral heterogeneity of cells from the adult muscle satellite cell niche. Cell.

[14] Zammit P.S., Relaix F., Nagata Y., Ruiz A.P., Collins C.A., Partridge T.A., Beauchamp J.R. (2006). Pax7 and myogenic progression in skeletal muscle satellite cells. J. Cell Sci..

[15] Seale P., Sabourin L.A., Girgis-Gabardo A., Mansouri A., Gruss P., Rudnicki M.A. (2000). Pax7 is required for the specification of myogenic satellite cells. Cell.

[16] Oustanina S., Hause G., Braun T. (2004). Pax7 directs postnatal renewal and propagation of myogenic satellite cells but not their specification. EMBO J..

[17] Von Maltzahn J., Jones A.E., Parks R.J., Rudnicki M.A. (2013). Pax7 is critical for the normal function of satellite cells in adult skeletal muscle. Proc. Natl. Acad. Sci. USA.

[18] Sincennes M.C., Brun C.E., Lin A.Y.T., Rosembert T., Datzkiw D., Saber J., Ming H., Kawabe Y.I., Rudnicki M.A. (2021). Acetylation of PAX7 controls muscle stem cell self-renewal and differentiation potential in mice. Nat. Commun..

[19] Ortuste Quiroga H.P., Fujimaki S., Ono Y. (2023). Pax7 reporter mouse models: A pocket guide for satellite cell research. Eur. J. Transl. Myol..

[20] Liu L., Cheung T.H., Charville G.W., Rando T.A. (2015). Isolation of skeletal muscle stem cells by fluorescence-activated cell sorting. Nat. Protoc..

[21] Maesner C.C., Almada A.E., Wagers A.J. (2016). Established cell surface markers efficiently isolate highly overlapping populations of skeletal muscle satellite cells by fluorescence-activated cell sorting. Skelet. Muscle.

[22] Tierney M.T., Stec M.J., Rulands S., Simons B.D., Sacco A. (2018). Muscle Stem Cells Exhibit Distinct Clonal Dynamics in Response to Tissue Repair and Homeostatic Aging. Cell Stem Cell.

[23] Scaramozza A., Park D., Kollu S., Beerman I., Sun X., Rossi D.J., Lin C.P., Scadden D.T., Crist C., Brack A.S. (2019). Lineage Tracing Reveals a Subset of Reserve Muscle Stem Cells Capable of Clonal Expansion under Stress. Cell Stem Cell.

[24] Garcia-Prat L., Perdiguero E., Alonso-Martin S., Dell’Orso S., Ravichandran S., Brooks S.R., Juan A.H., Campanario S., Jiang K., Hong X. (2020). FoxO maintains a genuine muscle stem-cell quiescent state until geriatric age. Nat. Cell Biol.

[25] Shi Y., He G., Lee W.C., McKenzie J.A., Silva M.J., Long F. (2017). Gli1 identifies osteogenic progenitors for bone formation and fracture repair. Nat. Commun..

[26] Peng J., Han L., Liu B., Song J., Wang Y., Wang K., Guo Q., Liu X., Li Y., Zhang J. (2023). Gli1 marks a sentinel muscle stem cell population for muscle regeneration. Nat. Commun..

[27] Barruet E., Garcia S.M., Striedinger K., Wu J., Lee S., Byrnes L., Wong A., Xuefeng S., Tamaki S., Brack A.S. (2020). Functionally heterogeneous human satellite cells identified by single cell RNA sequencing. eLife.

[28] Byun W.S., Lee J., Baek J.H. (2024). Beyond the bulk: Overview and novel insights into the dynamics of muscle satellite cells during muscle regeneration. Inflamm. Regen..

[29] Cho I.J., Lui P.P., Obajdin J., Riccio F., Stroukov W., Willis T.L., Spagnoli F., Watt F.M. (2019). Mechanisms, Hallmarks, and Implications of Stem Cell Quiescence. Stem Cell Rep..

[30] De Morree A., Rando T.A. (2023). Regulation of adult stem cell quiescence and its functions in the maintenance of tissue integrity. Nat. Rev. Mol. Cell Biol..

[31] Sousa-Victor P., García-Prat L., Muñoz-Cánoves P. (2022). Control of satellite cell function in muscle regeneration and its disruption in ageing. Nat. Rev. Mol. Cell Biol..

[32] Kuang S., Kuroda K., Le Grand F., Rudnicki M.A. (2007). Asymmetric self-renewal and commitment of satellite stem cells in muscle. Cell.

[33] Feige P., Brun C.E., Ritso M., Rudnicki M.A. (2018). Orienting Muscle Stem Cells for Regeneration in Homeostasis, Aging, and Disease. Cell Stem Cell.

[34] Fu X., Zhuang C.L., Hu P. (2022). Regulation of muscle stem cell fate. Cell Regen..

[35] Borycki A.G., Emerson C.P. (1997). Muscle determination: Another key player in myogenesis?. Curr. Biol..

[36] Han W.M., Mohiuddin M., Anderson S.E., Garcia A.J., Jang Y.C. (2019). Co-delivery of Wnt7a and muscle stem cells using synthetic bioadhesive hydrogel enhances murine muscle regeneration and cell migration during engraftment. Acta Biomater..

[37] Jiang S., Li H., Zhang L., Mu W., Zhang Y., Chen T., Wu J., Tang H., Zheng S., Liu Y. (2025). Generic Diagramming Platform (GDP): A comprehensive database of high-quality biomedical graphics. Nucleic Acids Res..

[38] Christ B., Ordahl C.P. (1995). Early stages of chick somite development. Anat. Embryol..

[39] Buckingham M., Bajard L., Chang T., Daubas P., Hadchouel J., Meilhac S., Montarras D., Rocancourt D., Relaix F. (2003). The formation of skeletal muscle: From somite to limb. J. Anat..

[40] Grefte S., Kuijpers-Jagtman A.M., Torensma R., Von den Hoff J.W. (2007). Skeletal muscle development and regeneration. Stem Cells Dev..

[41] Kassar-Duchossoy L., Giacone E., Gayraud-Morel B., Jory A., Gomès D., Tajbakhsh S. (2005). Pax3/Pax7 mark a novel population of primitive myogenic cells during development. Genes Dev..

[42] Esteves de Lima J., Relaix F. (2021). Master regulators of skeletal muscle lineage development and pluripotent stem cells differentiation. Cell Regen..

[43] Wosczyna M.N., Rando T.A. (2018). A Muscle Stem Cell Support Group: Coordinated Cellular Responses in Muscle Regeneration. Dev. Cell.

[44] Baht G.S., Bareja A., Lee D.E., Rao R.R., Huang R., Huebner J.L., Bartlett D.B., Hart C.R., Gibson J.R., Lanza I.R. (2020). Meteorin-like facilitates skeletal muscle repair through a Stat3/IGF-1 mechanism. Nat. Metab..

[45] Haroon M., Klein-Nulend J., Bakker A.D., Jin J., Seddiqi H., Offringa C., De Wit G.M.J., Le Grand F., Giordani L., Liu K.J. (2021). Myofiber stretch induces tensile and shear deformation of muscle stem cells in their native niche. Biophys. J..

[46] Tsuchiya Y., Kitajima Y., Masumoto H., Ono Y. (2020). Damaged Myofiber-Derived Metabolic Enzymes Act as Activators of Muscle Satellite Cells. Stem Cell Rep..

[47] Wang C., Rabadan Ros R., Martinez-Redondo P., Ma Z., Shi L., Xue Y., Guillen-Guillen I., Huang L., Hishida T., Liao H.K. (2021). In vivo partial reprogramming of myofibers promotes muscle regeneration by remodeling the stem cell niche. Nat. Commun..

[48] Sciorati C., Rigamonti E., Manfredi A.A., Rovere-Querini P. (2016). Cell death, clearance and immunity in the skeletal muscle. Cell Death Differ..

[49] Lievens E., Klass M., Bex T., Derave W. (2020). Muscle fiber typology substantially influences time to recover from high-intensity exercise. J. Appl. Physiol..

[50] Leng M., Yang F., Zhao J., Xiong Y., Zhou Y., Zhao M., Jia S., Liu L., Zheng Q., Gan L. (2025). Mitophagy-mediated S1P facilitates muscle adaptive responses to endurance exercise through SPHK1-S1PR1/S1PR2 in slow-twitch myofibers. Autophagy.

[51] Liu J., Liang X., Zhou D., Lai L., Xiao L., Liu L., Fu T., Kong Y., Zhou Q., Vega R.B. (2016). Coupling of mitochondrial function and skeletal muscle fiber type by a miR-499/Fnip1/AMPK circuit. EMBO Mol. Med..

[52] Widrick J.J., Trappe S.W., Costill D.L., Fitts R.H. (1996). Force-velocity and force-power properties of single muscle fibers from elite master runners and sedentary men. Am. J. Physiol..

[53] Zhang Z., Lin S., Luo W., Ren T., Huang X., Li W., Zhang X. (2022). Sox6 Differentially Regulates Inherited Myogenic Abilities and Muscle Fiber Types of Satellite Cells Derived from Fast- and Slow-Type Muscles. Int. J. Mol. Sci..

[54] Zimowska M., Kasprzycka P., Bocian K., Delaney K., Jung P., Kuchcinska K., Kaczmarska K., Gladysz D., Streminska W., Ciemerych M.A. (2017). Inflammatory response during slow- and fast-twitch muscle regeneration. Muscle Nerve.

[55] Cappellari O., Cossu G. (2013). Pericytes in development and pathology of skeletal muscle. Circ. Res..

[56] Kruger-Genge A., Blocki A., Franke R.P., Jung F. (2019). Vascular Endothelial Cell Biology: An Update. Int. J. Mol. Sci..

[57] Uezumi A., Ito T., Morikawa D., Shimizu N., Yoneda T., Segawa M., Yamaguchi M., Ogawa R., Matev M.M., Miyagoe-Suzuki Y. (2011). Fibrosis and adipogenesis originate from a common mesenchymal progenitor in skeletal muscle. J. Cell Sci..

[58] Chapman M.A., Meza R., Lieber R.L. (2016). Skeletal muscle fibroblasts in health and disease. Differentiation.

[59] Klingler W., Jurkat-Rott K., Lehmann-Horn F., Schleip R. (2012). The role of fibrosis in Duchenne muscular dystrophy. Acta Myol..

[60] Zhou S., Zhang W., Cai G., Ding Y., Wei C., Li S., Yang Y., Qin J., Liu D., Zhang H. (2020). Myofiber necroptosis promotes muscle stem cell proliferation via releasing Tenascin-C during regeneration. Cell Res..

[61] Beltra M., Pin F., Costamagna D., Duelen R., Renzini A., Ballaro R., Garcia-Castillo L., Iannuzzi A., Moresi V., Coletti D. (2022). PGC-1alpha in the myofibers regulates the balance between myogenic and adipogenic progenitors affecting muscle regeneration. iScience.

[62] Webster M.T., Manor U., Lippincott-Schwartz J., Fan C.M. (2016). Intravital Imaging Reveals Ghost Fibers as Architectural Units Guiding Myogenic Progenitors during Regeneration. Cell Stem Cell.

[63] Verma M., Asakura Y., Wang X., Zhou K., Unverdi M., Kann A.P., Krauss R.S., Asakura A. (2024). Endothelial cell signature in muscle stem cells validated by VEGFA-FLT1-AKT1 axis promoting survival of muscle stem cell. eLife.

[64] Zhang J., Muri J., Fitzgerald G., Gorski T., Gianni-Barrera R., Masschelein E., D’Hulst G., Gilardoni P., Turiel G., Fan Z. (2020). Endothelial Lactate Controls Muscle Regeneration from Ischemia by Inducing M2-like Macrophage Polarization. Cell Metab..

[65] Kang X., Qian J., Shi Y.X., Bian X.T., Zhang L.D., Li G.M., Wang L.T., Zhao J., Dong Z.Y., Yang M.M. (2024). Exercise-induced Musclin determines the fate of fibro-adipogenic progenitors to control muscle homeostasis. Cell Stem Cell.

[66] Sastourne-Arrey Q., Mathieu M., Contreras X., Monferran S., Bourlier V., Gil-Ortega M., Murphy E., Laurens C., Varin A., Guissard C. (2023). Adipose tissue is a source of regenerative cells that augment the repair of skeletal muscle after injury. Nat. Commun..

[67] Yu Y., Su Y., Wang G., Lan M., Liu J., Garcia Martin R., Brandao B.B., Lino M., Li L., Liu C. (2024). Reciprocal communication between FAPs and muscle cells via distinct extracellular vesicle miRNAs in muscle regeneration. Proc. Natl. Acad. Sci. USA.

[68] Wang K., Yang J., An Y., Wang J., Tan S., Xu H., Dong Y. (2024). MST1/2 regulates fibro/adipogenic progenitor fate decisions in skeletal muscle regeneration. Stem Cell Rep..

[69] De Lima J.E., Blavet C., Bonnin M.A., Hirsinger E., Comai G., Yvernogeau L., Delfini M.C., Bellenger L., Mella S., Nassari S. (2021). Unexpected contribution of fibroblasts to muscle lineage as a mechanism for limb muscle patterning. Nat. Commun..

[70] Sicherer S.T., Venkatarama R.S., Grasman J.M. (2020). Recent Trends in Injury Models to Study Skeletal Muscle Regeneration and Repair. Bioengineering.

[71] Wang Y., Lu J., Liu Y. (2022). Skeletal Muscle Regeneration in Cardiotoxin-Induced Muscle Injury Models. Int. J. Mol. Sci..

[72] Kumar A., Hardy D., Besnard A., Latil M., Jouvion G., Briand D., Thépenier C., Pascal Q., Guguin A., Gayraud-Morel B. (2016). Comparative Study of Injury Models for Studying Muscle Regeneration in Mice. PLoS ONE.

[73] Marshall J.S., Warrington R., Watson W., Kim H.L. (2018). An introduction to immunology and immunopathology. Allergy Asthma Clin. Immunol..

[74] Workman C.J., Szymczak-Workman A.L., Collison L.W., Pillai M.R., Vignali D.A. (2009). The development and function of regulatory T cells. Cell. Mol. Life Sci..

[75] Bonilla F.A., Oettgen H.C. (2010). Adaptive immunity. J. Allergy Clin. Immunol..

[76] Kushioka J., Chow S.K., Toya M., Tsubosaka M., Shen H., Gao Q., Li X., Zhang N., Goodman S.B. (2023). Bone regeneration in inflammation with aging and cell-based immunomodulatory therapy. Inflamm. Regen..

[77] Prame Kumar K., Nicholls A.J., Wong C.H.Y. (2018). Partners in crime: Neutrophils and monocytes/macrophages in inflammation and disease. Cell Tissue Res..

[78] Wang X., Zhou L. (2023). The multifaceted role of macrophages in homeostatic and injured skeletal muscle. Front. Immunol..

[79] Ziemkiewicz N., Hilliard G., Pullen N.A., Garg K. (2021). The Role of Innate and Adaptive Immune Cells in Skeletal Muscle Regeneration. Int. J. Mol. Sci..

[80] Yahara Y., Ma X., Gracia L., Alman B.A. (2021). Monocyte/Macrophage Lineage Cells From Fetal Erythromyeloid Progenitors Orchestrate Bone Remodeling and Repair. Front. Cell. Dev. Biol..

[81] Larouche J.A., Fraczek P.M., Kurpiers S.J., Yang B.A., Davis C., Castor-Macias J.A., Sabin K., Anderson S., Harrer J., Hall M. (2022). Neutrophil and natural killer cell imbalances prevent muscle stem cell-mediated regeneration following murine volumetric muscle loss. Proc. Natl. Acad. Sci. USA.

[82] Graca F.A., Stephan A., Minden-Birkenmaier B.A., Shirinifard A., Wang Y.D., Demontis F., Labelle M. (2023). Platelet-derived chemokines promote skeletal muscle regeneration by guiding neutrophil recruitment to injured muscles. Nat. Commun..

[83] Hoeffel G., Debroas G., Roger A., Rossignol R., Gouilly J., Laprie C., Chasson L., Barbon P.V., Balsamo A., Reynders A. (2021). Sensory neuron-derived TAFA4 promotes macrophage tissue repair functions. Nature.

[84] Lu Y.Z., Nayer B., Singh S.K., Alshoubaki Y.K., Yuan E., Park A.J., Maruyama K., Akira S., Martino M.M. (2024). CGRP sensory neurons promote tissue healing via neutrophils and macrophages. Nature.

[85] Miyamoto Y., Kikuta J., Matsui T., Hasegawa T., Fujii K., Okuzaki D., Liu Y.C., Yoshioka T., Seno S., Motooka D. (2024). Periportal macrophages protect against commensal-driven liver inflammation. Nature.

[86] Shang M., Cappellesso F., Amorim R., Serneels J., Virga F., Eelen G., Carobbio S., Rincon M.Y., Maechler P., De Bock K. (2020). Macrophage-derived glutamine boosts satellite cells and muscle regeneration. Nature.

[87] Ratnayake D., Nguyen P.D., Rossello F.J., Wimmer V.C., Tan J.L., Galvis L.A., Julier Z., Wood A.J., Boudier T., Isiaku A.I. (2021). Macrophages provide a transient muscle stem cell niche via NAMPT secretion. Nature.

[88] He Y., Heng Y., Qin Z., Wei X., Wu Z., Qu J. (2023). Intravital microscopy of satellite cell dynamics and their interaction with myeloid cells during skeletal muscle regeneration. Sci. Adv..

[89] Babaeijandaghi F., Cheng R., Kajabadi N., Soliman H., Chang C.K., Smandych J., Tung L.W., Long R., Ghassemi A., Rossi F.M.V. (2022). Metabolic reprogramming of skeletal muscle by resident macrophages points to CSF1R inhibitors as muscular dystrophy therapeutics. Sci. Transl. Med..

[90] Krasniewski L.K., Chakraborty P., Cui C.Y., Mazan-Mamczarz K., Dunn C., Piao Y., Fan J., Shi C., Wallace T., Nguyen C. (2022). Single-cell analysis of skeletal muscle macrophages reveals age-associated functional subpopulations. eLife.

[91] Sousa N.S., Bica M., Bras M.F., Sousa A.C., Antunes I.B., Encarnacao I.A., Costa T.M., Martins I.B., Barbosa-Morais N.L., Sousa-Victor P. (2024). The immune landscape of murine skeletal muscle regeneration and aging. Cell Rep..

[92] Burzyn D., Kuswanto W., Kolodin D., Shadrach J.L., Cerletti M., Jang Y., Sefik E., Tan T.G., Wagers A.J., Benoist C. (2013). A special population of regulatory T cells potentiates muscle repair. Cell.

[93] Zhang J., Xiao Z., Qu C., Cui W., Wang X., Du J. (2014). CD8 T cells are involved in skeletal muscle regeneration through facilitating MCP-1 secretion and Gr1(high) macrophage infiltration. J. Immunol..

[94] Kuswanto W., Burzyn D., Panduro M., Wang K.K., Jang Y.C., Wagers A.J., Benoist C., Mathis D. (2016). Poor Repair of Skeletal Muscle in Aging Mice Reflects a Defect in Local, Interleukin-33-Dependent Accumulation of Regulatory T Cells. Immunity.

[95] Yamaguchi Y., Mann D.M., Ruoslahti E. (1990). Negative regulation of transforming growth factor-beta by the proteoglycan decorin. Nature.

[96] Zhang X., Cao D., Xu L., Xu Y., Gao Z., Pan Y., Jiang M., Wei Y., Wang L., Liao Y. (2023). Harnessing matrix stiffness to engineer a bone marrow niche for hematopoietic stem cell rejuvenation. Cell Stem Cell.

[97] Hynes R.O. (2009). The extracellular matrix: Not just pretty fibrils. Science.

[98] Ricard-Blum S. (2011). The collagen family. Cold Spring Harb. Perspect. Biol..

[99] Mavropalias G., Boppart M., Usher K.M., Grounds M.D., Nosaka K., Blazevich A.J. (2023). Exercise builds the scaffold of life: Muscle extracellular matrix biomarker responses to physical activity, inactivity, and aging. Biol. Rev. Camb. Philos. Soc..

[100] Lukjanenko L., Jung M.J., Hegde N., Perruisseau-Carrier C., Migliavacca E., Rozo M., Karaz S., Jacot G., Schmidt M., Li L. (2016). Loss of fibronectin from the aged stem cell niche affects the regenerative capacity of skeletal muscle in mice. Nat. Med..

[101] Martins S.G., Ribeiro V., Melo C., Paulino-Cavaco C., Antonini D., Dayalan Naidu S., Murtinheira F., Fonseca I., Saget B., Pita M. (2024). Laminin-α2 chain deficiency in skeletal muscle causes dysregulation of multiple cellular mechanisms. Life Sci. Alliance.

[102] Mousavizadeh R., Hojabrpour P., Eltit F., McDonald P.C., Dedhar S., McCormack R.G., Duronio V., Jafarnejad S.M., Scott A. (2020). β1 integrin, ILK and mTOR regulate collagen synthesis in mechanically loaded tendon cells. Sci. Rep..

[103] Adair-Kirk T.L., Atkinson J.J., Broekelmann T.J., Doi M., Tryggvason K., Miner J.H., Mecham R.P., Senior R.M. (2003). A site on laminin alpha 5, AQARSAASKVKVSMKF, induces inflammatory cell production of matrix metalloproteinase-9 and chemotaxis. J. Immunol..

[104] Sarate R.M., Hochstetter J., Valet M., Hallou A., Song Y., Bansaccal N., Ligare M., Aragona M., Engelman D., Bauduin A. (2024). Dynamic regulation of tissue fluidity controls skin repair during wound healing. Cell.

[105] Gillies A.R., Lieber R.L. (2011). Structure and function of the skeletal muscle extracellular matrix. Muscle Nerve.

[106] Meyer G.A., Lieber R.L. (2011). Elucidation of extracellular matrix mechanics from muscle fibers and fiber bundles. J. Biomech..

[107] Urciuolo A., Quarta M., Morbidoni V., Gattazzo F., Molon S., Grumati P., Montemurro F., Tedesco F.S., Blaauw B., Cossu G. (2013). Collagen VI regulates satellite cell self-renewal and muscle regeneration. Nat. Commun..

[108] Metti S., Da Ros F., Toniato G., Cescon M., Bonaldo P. (2024). Native collagen VI delays early muscle stem cell differentiation. J. Cell Sci..

[109] Van Ry P.M., Minogue P., Hodges B.L., Burkin D.J. (2014). Laminin-111 improves muscle repair in a mouse model of merosin-deficient congenital muscular dystrophy. Hum. Mol. Genet..

[110] Yao Y., Norris E.H., Mason C.E., Strickland S. (2016). Laminin regulates PDGFRbeta(+) cell stemness and muscle development. Nat. Commun..

[111] Rayagiri S.S., Ranaldi D., Raven A., Mohamad Azhar N.I.F., Lefebvre O., Zammit P.S., Borycki A.G. (2018). Basal lamina remodeling at the skeletal muscle stem cell niche mediates stem cell self-renewal. Nat. Commun..

[112] Stearns-Reider K.M., Hicks M.R., Hammond K.G., Reynolds J.C., Maity A., Kurmangaliyev Y.Z., Chin J., Stieg A.Z., Geisse N.A., Hohlbauch S. (2023). Myoscaffolds reveal laminin scarring is detrimental for stem cell function while sarcospan induces compensatory fibrosis. NPJ Regen. Med..

[113] Hayward M.K., Muncie J.M., Weaver V.M. (2021). Tissue mechanics in stem cell fate, development, and cancer. Dev. Cell.

[114] Ladoux B., Mege R.M. (2017). Mechanobiology of collective cell behaviours. Nat. Rev. Mol. Cell Biol..

[115] Mao Y., Wickstrom S.A. (2024). Mechanical state transitions in the regulation of tissue form and function. Nat. Rev. Mol. Cell Biol..

[116] Lemke S.B., Schnorrer F. (2017). Mechanical forces during muscle development. Mech. Dev..

[117] Vining K.H., Mooney D.J. (2017). Mechanical forces direct stem cell behaviour in development and regeneration. Nat. Rev. Mol. Cell Biol..

[118] Tao J., Choudhury M.I., Maity D., Kim T., Sun S.X., Fan C.M. (2023). Mechanical compression creates a quiescent muscle stem cell niche. Commun. Biol..

[119] Ma N., Chen D., Lee J.H., Kuri P., Hernandez E.B., Kocan J., Mahmood H., Tichy E.D., Rompolas P., Mourkioti F. (2022). Piezo1 regulates the regenerative capacity of skeletal muscles via orchestration of stem cell morphological states. Sci. Adv..

[120] Hirano K., Tsuchiya M., Shiomi A., Takabayashi S., Suzuki M., Ishikawa Y., Kawano Y., Takabayashi Y., Nishikawa K., Nagao K. (2023). The mechanosensitive ion channel PIEZO1 promotes satellite cell function in muscle regeneration. Life Sci. Alliance.

[121] Mohyeldin A., Garzon-Muvdi T., Quinones-Hinojosa A. (2010). Oxygen in stem cell biology: A critical component of the stem cell niche. Cell Stem Cell.

[122] Brahimi-Horn M.C., Pouysségur J. (2007). Oxygen, a source of life and stress. FEBS Lett..

[123] Liu W., Wen Y., Bi P., Lai X., Liu X.S., Liu X., Kuang S. (2012). Hypoxia promotes satellite cell self-renewal and enhances the efficiency of myoblast transplantation. Development.

[124] Redshaw Z., Loughna P.T. (2012). Oxygen concentration modulates the differentiation of muscle stem cells toward myogenic and adipogenic fates. Differentiation.

[125] Drouin G., Couture V., Lauzon M.A., Balg F., Faucheux N., Grenier G. (2019). Muscle injury-induced hypoxia alters the proliferation and differentiation potentials of muscle resident stromal cells. Skelet. Muscle.

[126] Mao Y., Zhang J., Zhou Q., He X., Zheng Z., Wei Y., Zhou K., Lin Y., Yu H., Zhang H. (2024). Hypoxia induces mitochondrial protein lactylation to limit oxidative phosphorylation. Cell Res..

